# Allometric scaling of a superposition eye optimizes sensitivity and acuity in large and small hawkmoths

**DOI:** 10.1098/rspb.2022.0758

**Published:** 2022-07-27

**Authors:** Anna Stöckl, Rebecca Grittner, Gavin Taylor, Christoph Rau, Andrew J. Bodey, Almut Kelber, Emily Baird

**Affiliations:** ^1^ Behavioral Physiology and Sociobiology (Zoology II), University of Würzburg, Würzburg, Germany; ^2^ Institute for Globally Distributed Open Research and Education (IGDORE), Ribeirão Preto, Brazil; ^3^ Diamond Light Source, Harwell Science and Innovation Campus, Didcot, UK; ^4^ Department of Biology, Lund University, Lund, Sweden; ^5^ Department of Zoology, Stockholm University, Stockholm, Sweden

**Keywords:** vision, allometry, sensitivity, acuity, insect, eye morphology

## Abstract

Animals vary widely in body size within and across species. This has consequences for the function of organs and body parts in both large and small individuals. How these scale, in relation to body size, reveals evolutionary investment strategies, often resulting in trade-offs between functions. Eyes exemplify these trade-offs, as they are limited by their absolute size in two key performance features: sensitivity and spatial acuity. Due to their size polymorphism, insect compound eyes are ideal models for studying the allometric scaling of eye performance. Previous work on apposition compound eyes revealed that allometric scaling led to poorer spatial resolution and visual sensitivity in small individuals, across a range of insect species. Here, we used X-ray microtomography to investigate allometric scaling in superposition compound eyes—the second most common eye type in insects—for the first time. Our results reveal a novel strategy to cope with the trade-off between sensitivity and spatial acuity, as we show that the eyes of the hummingbird hawkmoth retain an optimal balance between these performance measures across all body sizes.

## 1. Introduction

Animals of the same species can vary considerably in body size [1–3]. Such differences have performance consequences for body parts or organs in larger and smaller individuals, particularly when their function depends on absolute rather than relative size [4]. A key organ that exemplifies the evolutionary strategies to cope with the behavioural and ecological consequences of body size variation is the eye, because eyes are performance-constrained by their absolute size. Eye size, in turn, is limited by body size, due to the energy and weight constraints associated with carrying large eye structure, particularly in small flying animals [5]. Eye size limits two central features of eye functionality: sensitivity and spatial resolution [6–9]. Larger eyes can collect more photons, due to a potentially larger light-collecting aperture and focal length, as well as the diameter and length of their photoreceptive units. Higher sensitivity is not just important for seeing well in dim light [9], but also for discriminating fine contrast changes at higher light intensities [7,8]. In addition, spatial resolution is limited by the number of visual units packed into an eye of a given viewing angle—thus the number of ‘pixels’ that can be resolved across the eyes' field of view [6–9]. While a small eye could densely pack many visual units with high acuity, the small eye size means that they will have to be narrower than in larger eyes, and thus of lower light sensitivity, and consequently lower contrast resolution [7,8]. This size limit on spatial resolution is exacerbated in eyes with small lenses, such as the compound eyes of insects. Here, the small diameter of facets can set a diffraction limit to the optical resolution, resulting in blurred visual projections [10–13]. Combined with their generally small body size that restricts the absolute eye size [5], these challenges to sensitivity and spatial resolution make insect compound eyes an ideal model to study how eyes scale allometrically for optimal performance in small animals.

One strategy that most insect species use to cope with these challenges is to preserve an eye as large as possible in small individuals, resulting in a negative allometric relationship between eye and body size. This means that smaller individuals have absolutely smaller but relatively larger eyes for their body size within and across species (bees [14–17], ants [18,19], butterflies [20,21] and flies [22]). Positive allometry between eye and body size is rare [23]. A second trend commonly observed in insects is negative allometry between facet size and eye size [17–20,22]. A relatively larger facet size in smaller individuals can improve visual sensitivity [6]. Larger bumblebees, for example, forage at lower light intensities than smaller ones [24] and detect smaller point-targets because of an increased sensitivity of individual ommatidia [15]. These scaling strategies do not always manifest over the entire eye, but can also differ locally [25]. In bumblebees, larger individuals benefit from optimizing spatial acuity in their frontal acute zone, while the overall spatial resolution of the eye remains similar across individual body sizes [17].

These insights into the scaling strategies of insect eyes are based on apposition compound eyes, in which the sensitivity of individual optical units is limited by their facet size. A large proportion of insects, however, especially among the Lepidoptera and Coleoptera [26,27], possess a different eye type: superposition compound eyes. This eye type is typically found in nocturnal insects, though with prominent diurnal exceptions. It provides a highly increased sensitivity compared to apposition eyes [6,7,9] since hundreds of neighbouring facets can focus light onto a single rhabdom, acting as a functional lens with an aperture larger than that of a single facet [26]. This increased single-ommatidial photon capture might lead to different selection constraints in the scaling with body size compared to apposition eyes [28]. Moreover, because of the intricate optical arrangements of multiple corneal lenses and crystalline cones that focus light onto a single rhabdom, superposition compound eyes might be less flexible for local modifications, as these could compromise the superposition optics. Thus, revealing the scaling strategies of superposition compound eyes will be an important contribution to understanding the visual constraints of many beetle and moths species—many of which are important diurnal and nocturnal pollinators [29,30].

To quantify how superposition compound eyes scale with body size, we chose to study an insect model that can directly be compared to species with apposition eyes: the hummingbird hawkmoth *Macroglossum stellatarum*. As day-active nectar foragers [31], these moths are under similar visual constraints as many previously tested hymenopteran and lepidopteran species, and share habitats and host plants with common Eurasian bee and butterfly species. To quantify the allometric scaling of optical and sensory structures of the eyes of large and small hummingbird hawkmoths, we used X-ray micro-computed tomography [17,32,33]. Even though the eyes of hawkmoths are generally designed for high photon catch, we found strong negative allometry between eye and body size, and between facet diameter and eye size, resulting in a proportional increase of sensitivity in small hawkmoth eyes. Our modelling provides an explanation for this finding: the relatively increased facet diameters decreased the amount of diffraction blur, thus benefiting spatial acuity in small eyes. Moreover, the observed scaling exponents optimized the eyes of large and small individuals to the smallest possible variation in sensitivity and spatial acuity, thus retaining a stable optical system across scales. Our results thus demonstrate that both visual functions are mutually optimized by scaling strategies in small superposition compound eyes.

## 2. Methods

**Figure 1.**
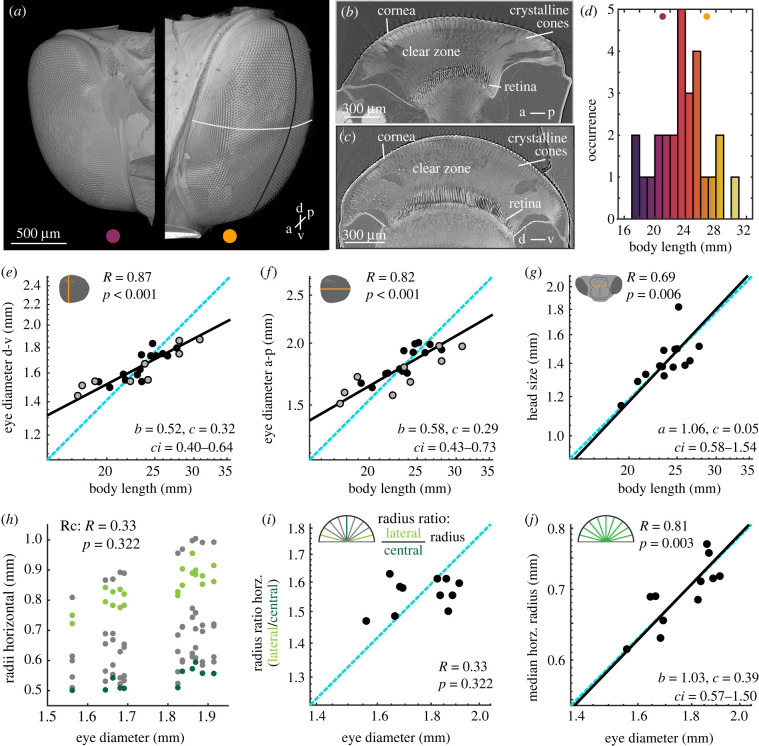
Allometric scaling of eye and head size in *Macroglossum stellatarum*. (*a*) X-ray microtomography images of two individuals of *M. stellatarum*. The animal to the left had a body length of 21.8 mm, the right one of 26.5 mm. The scale bar applies to both eyes. The dot colours refer to the body size of the animals from which the eyes were sourced (also shown in (*d*)). (*b*) Representative horizontal and (c) vertical section through the centre of the eye (see white and black lines in (*a*)) with the cornea, crystalline cones, clear zone and retina indicated. (*d*) Body length of the individuals selected for this study. Allometric scaling of the (*e*) dorsal-ventral, (*f*) the anterior-posterior diameter of the eye, and (*g*) the head size measured from the left to the right base of the mouth parts (electronic supplementary material, figure S2A). (*h*) To test whether the shape of the eye differed across eye diameters, we measured the distance from the nodal point formed by the edges of the cornea to the corneal surface for nine evenly spaced radii in horizontal sections (see electronic supplementary material, figure S3D–F for frontal ones, see sketch in (*i*) for colour code). (*i*) We calculated the ratio between the average lateral radii (light green) and the central radius (dark green) as a proxy for the cornea's shape, and assessed its allometric scaling. (*j*) The allometric scaling of the median of the central seven radius measurements (green) with eye diameter. (*e–g,i,j*) Data from individual hawkmoths was measured by either X-ray microtomography (black dots), or light-microscopy (grey dots). The dashed cyan line indicates isometric scaling and the black line represents the allometric scaling relationship. *R* is the Pearson correlation coefficient of the log-transformed data, and *p* denotes its statistical significance. Given the significant linear correlations in (*e–g,j*), the allometric relationship was calculated using reduced major axis regression, with the exponential scaling exponent *b*, the normalization constant *c,* and the confidence interval *ci* of *b*. (Online version in colour.)

**Figure 2.**
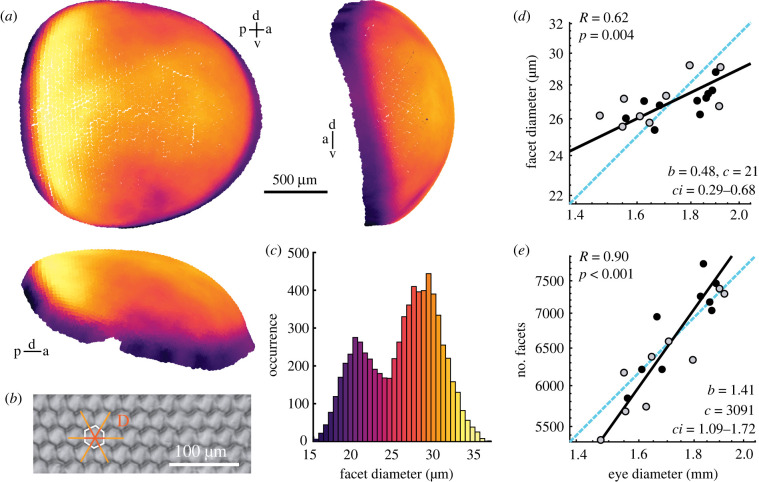
Cornea morphology and facet allometry of *Macroglossum stellatarum.* (*a*) three-dimensional reconstruction of the facets of an example eye of *M. stellatarum* (total facets: 7111), with facet diameter indicated by the colour scale in (*c*), top left: sagittal view, top right: anterior view, bottom: dorsal view. (*b*) The facet diameter was calculated as the average of 3 measurements across 3 facets each (in light orange), centred on the facet of interest and then divided by the number of facets spanned. (*c*) Facet diameter distribution of all facets of the eye in (*c*). (*d*) Allometric scaling of the median facet diameter of the eyes' main facets (greater than 20 µm), and (*e*) the total number of facets with eye diameter. The total facet number was estimated by dividing the surface of the eye (approximated by a circular area based on the eye diameter) by the median facet size. (*d,e*) Data from individual hawkmoths was measured by either X-ray microtomography (black dots), or light-microscopy (grey dots). The dashed cyan line indicates isometric scaling and the black line represents the allometric scaling relationship. *R* is the Pearson correlation coefficient of the log-transformed data, and *p* denotes its statistical significance. Given the significant linear correlations in (*d*) and (*e*), the allometric relationship was calculated using reduced major axis regression, with the exponential scaling exponent *b*, the normalization constant *c,* and the confidence interval *ci* of *b*. (Online version in colour.)

To study how the eye size and eye morphology differed with body size in the superposition compound eye of *Macroglossum stellatarum* (figure 1*a*), we selected a total of 25 individuals with a wide range of body sizes (figure 1*d*). We obtained surface measures of their eyes (eye diameter: figure 1*b,c*, facet size: figure 2*d*) from light microscopy (9 animals) and X-ray microtomography (16 animals), which we combined in the subsequent analysis. We relied on the X-ray tomography data for parameters requiring optical sections. For detailed descriptions of our methods, see electronic supplementary material, 'Methods'.

## 3. Results

### (a) Eye size scales negatively allometric with body size

We observed significant negative allometry between eye diameter and body length with a scaling coefficient of 0.522 for the dorsoventral eye diameter (figure 1*e*), and 0.577 for the anterior-posterior diameter (figure 1*f*). This indicated that smaller hawkmoths had relatively larger eyes than bigger moths. Moreover, the two axes of the eye had a highly significant correlation, which scaled isometrically (electronic supplementary material, figure S3A), and allowed us to combine the eye diameter into a single measure where required by taking the average of the two measures. Since the eyes comprise a substantial portion of the hawkmoth head, we also checked whether the scaling in eye size was mirrored by scaling in head size. Since our specimen preparation did not preserve the entire head (see electronic supplementary material, 'Methods'), we measured proxies of head size using landmarks which could be reliably recognized in all preparations (electronic supplementary material, figure S2A): the lateral (figure 1*g*) and dorsoventral (electronic supplementary material, figure S2C) extent of the mouth-part base, and the dorsoventral extent of the head capsule surrounding the optic lobes of the brain (electronic supplementary material, figure S2B). All of these scaled isometrically with body size, indicating that only the eyes of *M. stellatarum*, not the head as a whole, scale negatively allometrically with body size.

### (b) Smaller animals have relatively larger, but fewer facets

Given the overall negative allometric relationship between eye and body size, we next investigated how structures of the eye that relate to spatial acuity and visual sensitivity scale with body and eye size. To quantify the size of the corneal facet lenses (figure 2*a*), we labelled all facets in two eyes, and 60–70 regularly spaced facets in all other eyes (*n* = 19). The facet lenses varied in diameter across the hawkmoths’ eyes, with the largest facets being located in a median band along the anterior-posterior extent of the eye surface, and along the entire dorsoventral extent of the posterior part of the eye. The histogram of all facet lenses of a completely reconstructed corneal surface clearly showed two peaks (figure 2*c*, electronic supplementary material, figure S4A), representing the main facets of the eye and a ring of distinctly smaller facets located around the eyes' perimeter, which are covered by scales in intact hawkmoths. The median diameters of outer facets, which might be structural in nature, did not correlate significantly with eye diameter (electronic supplementary material, figure S4C). By contrast, the median diameters of the functional main facets (greater than 20 µm), correlated significantly with eye diameter (figure 2*d*) and body size (electronic supplementary material, figure S4B).

A negative allometric scaling of facet diameter to eye diameter would indicate that smaller animals have fewer facets relative to their eye diameter than large ones – provided that the relationship between the surface area of the cornea and eye size did not differ. Since the surface area depends on the shape of the cornea, we analysed the scaling of the cornea's curvature with eye diameter (figure 1*h–j*; electronic supplementary material, figure S3D-F). We calculated the ratio of the central and lateral radii of the eye in horizontal (figure 1*i*) and frontal sections (electronic supplementary material, figure S3E) at the dorsoventral and anterior-posterior median of the eye, respectively. There was no significant correlation of the curvature ratio with eye diameter (figure 1*i*; electronic supplementary material, figure S3E), while the average radius of the cornea scaled isometrically with eye size (figure 1*j*; electronic supplementary material, figure S3F), indicating that the corneas' curvature remained the same in large and small eyes. This confirmed the validity of our approach to estimate eye surface based on eye diameter. It also allowed us to estimate the total number of facets per eye, by dividing the eye surface area by the median facet diameter. The total facet number scaled positively allometric (figure 2*e*), with the lower-bound confidence interval exceeding isometry. Thus, smaller hummingbird hawkmoths invested in larger facet diameters at the cost of the total number of facets.

To assess whether the shape of the cornea differed with eye diameter, we measured evenly spaced eye radii in horizontal and vertical sections (figure 1*h*; electronic supplementary material, figure S3D). The ratio of the two lateral-most radii and the central one in each section was used as an indicator for shape: if, for example, a larger eye was rounder than a smaller one, the ratio would be smaller in larger eyes, while it would remain the same if the shape of the cornea did not change. We thus analysed the allometric scaling of the radius ratio with eye size and found there was no significant correlation in either the horizontal (figure 1*i*) or frontal sections (electronic supplementary material, figure S3E). The median of all radii in both horizontal and frontal sections scaled isometrically with eye diameter (figure 1*j*, electronic supplementary material, figure S3F).

### (c) Rhabdom distance, but not length, scales negatively isometric with eye size

**Figure 3.**
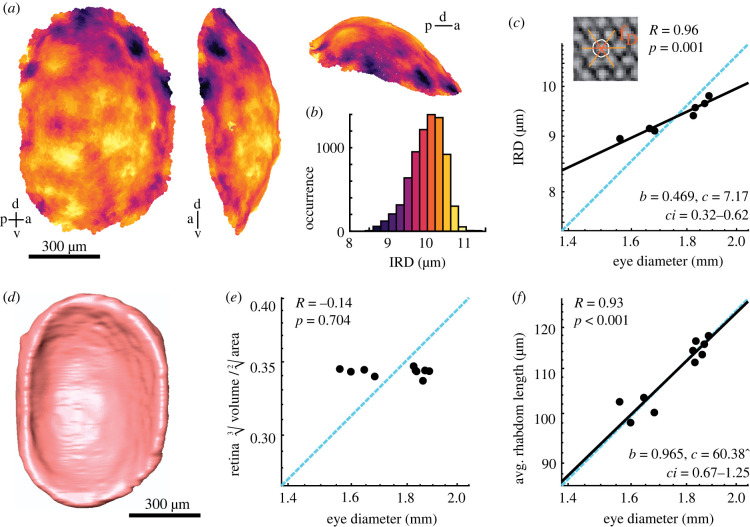
Retinal morphology and rhabdom scaling. (*a*) Three-dimensional reconstruction of the rhabdoms in an example retina (with 7560 rhabdoms), with inter-rhabdom distance (IRD) indicated by the colour scale in (*b*). From left to right: sagittal view, anterior view and dorsal view. (*c*) The IRD was measured as the average distance between rhabdoms as shown in the inset. Allometric scaling of the IRD. (*d*) Three-dimensional-reconstruction of the retinal volume of the example eye. (*e*) Differences in retinal shape assessed as the ratio between retinal volume and surface area across eye diameters. (*f*) A conserved retinal shape across eye sizes allowed us to use the thickness of the retina as a proxy for the average rhabdom length, calculated as the volume divided by half the surface area. (*c,e,f*) Data from individual hawkmoths was measured by X-ray microtomography (black dots, *n* = 7 in (*c*), *n* = 10 in (*e,f*) ). The dashed cyan line indicates isometric scaling and the black line represents the allometric scaling relationship. *R* is the Pearson correlation coefficient of the log-transformed data, and *p* denotes its statistical significance. Given the significant linear correlations in (*c,f*), the allometric relationship was calculated using reduced major axis regression, with the exponential scaling exponent *b*, the normalization constant *c,* and the confidence interval *ci* of *b*. (Online version in colour.)

We next analysed whether the scaling relationship of facet lenses transferred to the retina. In a typical apposition compound eye, each facet lens forms a structural unit with a group of photoreceptors (the rhabdom), termed an ommatidium. In most superposition compound eyes, the 1 : 1 relationship between facet lenses and photoreceptive units exists as well, although the optical relationship is uncoupled by the optical units in the superposition pupil focusing light from many facet lenses onto a single rhabdom [9,26]. In the hummingbird hawkmoth, the anatomical 1 : 1 relationship between facet lenses and retinal units was called into question, due to an optically measured inhomogeneity in facet diameter and retinal packing (Warrant [34]). Since the tracheal sheaths surrounding the photoreceptors [34] provided high optical contrast, we could fully reconstruct all rhabdom positions in two eyes (figure 3*a*). From this, we calculated inter-rhabdom distances (IDR, figure 3*b*) similar to the inter-facet distances (figure 2*a*). The inter-facet and inter-rhabdom distances showed very different local patterns across the eye, highlighting that the facet distribution was uncoupled from the retinal one. However, the total number of rhabdoms and facets identified in two eyes were very similar. Indeed, the number of rhabdoms was 6% and 8% higher – a divergence probably caused by an underestimation of the number of facets, as some of the structural facets could not be resolved. The IRDs of the fully reconstructed retinas showed only a single-peaked distribution (figure 3*b*), as compared to the double-peaked distribution of the facet sizes (figure 2*c*). Nevertheless, there was still considerable variation in the IRDs, which was systematically larger in the ventral than the dorsal half of the retina (figure 3*a*).

For all eyes, we determined the average IRDs in the centre of the retina (see electronic supplementary material, 'Methods') as a measure for the separation of the anatomical sampling base of the eyes. The average IRD scaled negatively allometric with eye size across individuals (figure 3*c*), indicating that smaller individuals had distinctly larger IRDs than expected for their eye size. Moreover, IRDs scaled with the same coefficient as facet diameter across eye size (figure 2*d*), and indeed there was a linear relationship between IRDs and facet diameters (electronic supplementary material, figure S6C), giving additional support to the notion that the number of photoreceptor units in the retina matches the number of facets in the cornea.

To assess how rhabdom length scaled with eye size, we used the thickness of the retina as a proxy. This is possible if the retinal shape was the same in animals of different body size. We confirmed this by comparing the ratio of retinal volume and surface area across eye sizes: if the retina became flatter with eye size, the ratio should decrease, while it should increase if the retina became thicker. Since the ratio remained the same across eye size (figure 3*e*), we concluded that retinal shape did not scale with eye size. We thus estimated the rhabdom length by dividing the retinal volume by half its surface area. Unlike IRD, rhabdom length scaled isometrically with eye size (figure 3*f*). Thus, smaller hummingbird hawkmoths invested in larger IRDs at the cost of total number of rhabdoms, while the length of their rhabdoms scaled isometrically with size.

### (d) Both sensitivity and spatial acuity are optimized in small hawkmoths

**Figure 4.**
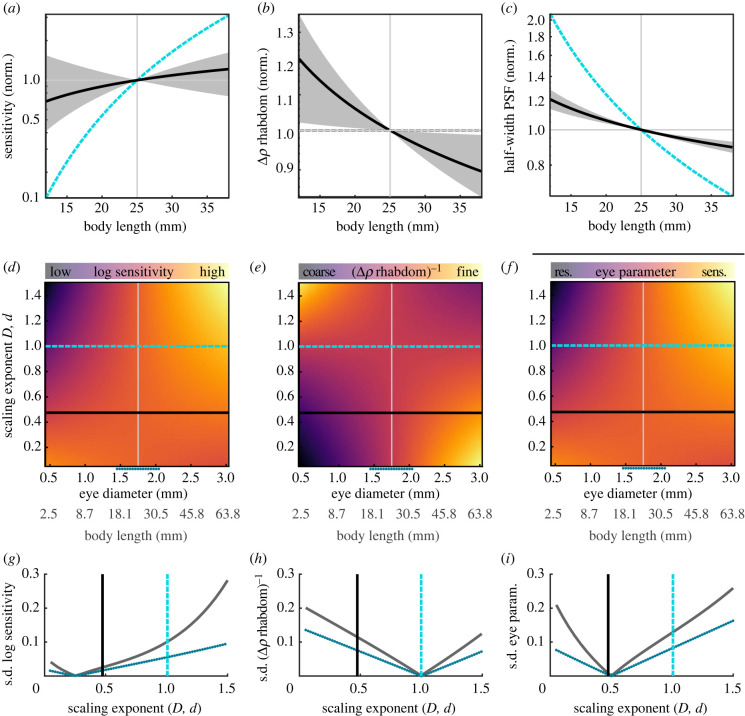
Model estimation of the allometry of spatial acuity and sensitivity. We used the measured allometric relations of the inter-facet and inter-rhabdom distance *D* and *d*, rhabdom length *l* and focal length *f* (the latter two scaling isometrically) to calculate (*a*) the sensitivity of a single ommatidium according to [35], (*b*) the rhabdom acceptance angle and (*c*) half-width of the point spread function (PSF), according to [6]. All estimates of eye performance were calculated for body lengths ranging from 12.5 mm to 37.5 mm and normalized to a median-sized animal of 25 mm body length. All calculations were compared to estimates based on an eye in which all parameters scaled isometrically (cyan line). The confidence intervals were computed by applying the same calculations to the scaling parameters (exponent and *y*-axis intercept) with added and subtracted confidence intervals obtained from the regression analysis. (*d*) Log-transformed sensitivity, (*e*) rhabdom acceptance angle, and (*f*) eye parameter were calculated for a range of scaling exponents applied to the inter-facet and inter-rhabdom distance *D* and *d* (see electronic supplementary material, 'Methods'). (*d–f*) The resulting values are depicted for different eye diameters (with corresponding body lengths indicated in grey), normalized to the highest sensitivity, smallest rhabdom acceptance angle, and largest eye parameter. The measured scaling exponent is indicated by the black line, and isometry by the blue dashed line. The dotted blue line below the *x*-axis indicates the measured size variation. Variation in (*g*) log sensitivity, (*h*) rhabdom acceptance angle, and (*i*) eye parameter for a given scaling exponent across eye sizes, quantified as the standard deviation (s.d.) for the entire range of eye diameters (grey line), and the measured range (blue dotted line). The black line indicates the measured exponents, and the blue dashed line isometry. (Online version in colour.)

To understand how the scaling of the optical and sensory structures affected the function of large and small hawkmoth eyes, we used the observed allometric relations to calculate key performance measures of eyes: single-ommatidium sensitivity (figure 4*a*; electronic supplementary material, 'Methods', equation 5, according to [36]), spatial resolution as the photoreceptor acceptance angle (figure 4*b*; electronic supplementary material, 'Methods', equation 4, according to [6]), and the limiting feature of spatial acuity: the half-width of the Airy disc (figure 4*c*; electronic supplementary material, 'Methods', equation 3). To do so, we used the measured scaling coefficients of the facet diameter, inter-rhabdom distance (IRD), and rhabdom length to estimate eye performance for animals of different body lengths. We approximated the scaling of rhabdom diameters by the scaling of the IRD, assuming that the tracheal sheath surrounding each rhabdom (which contributed to the IRD), scales isometrically with eye size and remains constant across the eye, which electron microscopic sections support [34]. Furthermore, for these calculations, the focal length of the eye was required. Although it cannot be directly determined anatomically in aspherical superposition compound eyes [36], we show that it is valid to apply the same scaling coefficient for the focal length as for the eye diameter (see electronic supplementary material, 'Methods').

Given these scaling parameters, we could show that the sensitivity of a single ommatidium scaled with distinct negative allometry compared to an eye in which all structures scaled isometrically (figure 4*a*): with isometric scaling, each ommatidium of an animal with 12.5 mm body length would have a ten times reduced sensitivity compared to an animal with 25 mm body length (the median). The reduction in sensitivity given the measured scaling was only 30%, and thus seven times higher than for isometric scaling. Moreover, the 95% confidence intervals still included the sensitivity value of the median sized animal, indicating that there is a negligible difference in sensitivity between animals differing in size by a factor of 2.

For the estimate of spatial resolution, our model showed that the photoreceptor acceptance angle of animals of 12.5 mm body was 20% larger compared to the median animal of 25 mm body length – with confidence intervals not overlapping the median (figure 4*b*). This represented a distinct difference from isometric scaling, which did not predict any differences from a median sized animal, because both the rhabdom diameter and focal length scaled isometrically in this case. The optical limitation of spatial resolution, the half-width of the fundamental mode of the point spread function (PSF) which causes diffraction at a single facet lens (see electronic supplementary material, 'Methods', equation 4), scaled so that smaller eyes had a relatively smaller diffraction blur circle than they would have had with isometric scaling (Airy disc, figure 4*c*).

### (e) The scaling of facets and rhabdoms minimized differences in the eyes’ optical function across body sizes

We next assessed how the observed scaling exponents of the inter-facet and rhabdom distance determined the performance of eyes across body sizes, compared to a range of hypothetical scaling exponents representing negative and positive allometry, as well as isometry. We focused on these two structures because they diverged strongly from isometry with eye size and scaled with very similar exponents so that a common exponent could be assumed for modelling (0.48 for facet diameter, 0.47 for rhabdom diameter, average of 0.475 indicated as the black line in figure 4*d–f*). The focal and rhabdom lengths, which also contribute to the acuity and sensitivity of the eye, scaled isometrically with eye size. We calculated the ommatidial sensitivity and rhabdom acceptance angle as before, across a range of possible allometric scaling parameters for a range of eye sizes (figure 4*d,e*). We also performed this calculation for the eye parameter (figure 4*f*; electronic supplementary material, 'Methods', equation 6), a measure of the eyes' optimization for sensitivity or spatial acuity (smaller values suggest optimization for acuity, large values for sensitivity).

For ommatidial sensitivity, isometric or positive allometric scaling resulted in distinctly higher sensitivity in larger than in smaller eyes (figure 4*d*). This strong divergence decreased down to a scaling exponent of approximately 0.3, below which the sensitivity was moderately higher in smaller than larger eyes. The measured scaling exponent (the black dashed line in figure 4*d–f*) yielded a moderate difference in sensitivity across eye sizes, as also described in figure 4*a*. A very different performance for small and large eyes was obtained for the rhabdom acceptance angle, where larger animals would have coarser angles than smaller ones for a scaling exponent above 1, and vice versa below 1. The same acceptance angle was predicted for all eye sizes with isometric scaling (figure 4*e*). Finally, the eye parameter, flipped in its effect for smaller and larger eyes at scaling exponents close to those measured in hawkmoth eyes (figure 4*f*): for scaling exponents higher than 0.5, larger eyes were optimized more strongly for sensitivity, while this was the case for smaller eyes for scaling exponents below 0.5. Across all three eye performance values, the scaling exponents observed in the eyes of *M. stellatarum* reduced the variance in sensitivity and eye parameter across eyes of different sizes compared to isometric scaling (figure 4*g*,*i*): the observed scaling exponents were close to the overall minimum of variance across eyes for sensitivity (figure 4*h*), while they fell right into the minimum for the eye parameter (figure 4*i*). This indicates that the scaling of facet and rhabdom diameters in the superposition compound eyes of hummingbird hawkmoths are optimized to reduce the variance in eye performance across eye and body sizes.

## 4. Discussion

In this study, we used three-dimensional X-ray microtomography to provide the first quantification of allometric scaling of the morphological and functional features of a superposition compound eye. We revealed that the overall scaling of the hummingbird hawkmoth's eye with body size was negatively allometric, as in many other insects. Even though the superposition optics provide generally higher sensitivity to light than the optics of apposition compound eyes of a similar size, we found that non-isometric scaling reduced the loss in sensitivity in the smaller eyes of smaller individuals even further. Overall, the allometric scaling of the hawkmoths' eye parameter minimizes differences in absolute sensitivity and spatial acuity across eye and body sizes.

### (a) Local inhomogeneities in hummingbird hawkmoth superposition eyes

To quantify the allometric scaling of hummingbird hawkmoth superposition eyes, we undertook the first three-dimensional structural characterization of these eyes, which revealed some unexpected features of their visual system. It has been described previously that, unusually for optical superposition compound eyes [26,28], hummingbird hawkmoth compound eyes are inhomogeneous [34]. Unlike the spherical eyes of their nocturnal relatives (for example *Deilephila elpenor* [37]), their cornea and retina are locally flattened, particularly in the anterior-posterior axis. Furthermore, facet and rhabdom diameters are inhomogeneously distributed across the eye (figures 2 and 3), reminiscent of the local acute zones in apposition compound eyes [17,38–40]. Our results confirmed previous data obtained using tissue sections of a band of increased facet diameter along the lateral midline of the eye [34]. In addition, we revealed that the largest facets in the hawkmoth eye are positioned at the posterior edge of the eye, extending over the entire dorsoventral axis. These facets were nearly 30% larger than the average facet diameter across the eye, suggesting that increased sensitivity in the posterior visual field is of high importance to the hawkmoths. This might serve to recognize approaching predators as early as possible, especially while hawkmoths are at their most vulnerable, hover-feeding from flowers [31,41]. Our data also provide evidence for two classes of facets in the eye of hummingbird hawkmoths: the main facets of the eye, and a group of distinctly smaller facets around its perimeter (figure 2*a*) that are covered in scales in intact hawkmoths. These two groups are visible as two clear peaks in the facet diameter histograms (figure 2*c*; electronic supplementary material, figure S4A). The fact that the small perimeter facets did not scale with eye size (electronic supplementary material, figure S4C), while main facets did (figure 2*d*), further suggests they are unlikely to be optically functional, but instead have a structural role. More research into the optical axes and focusing properties of these small facets will be required to elucidate whether they do play a functional or a purely structural role.

### (b) Anatomical existence of ommatidia in hummingbird hawkmoth eyes

Our results provide new context to previous anatomical findings from hummingbird hawkmoth eyes, which suggest that these eyes lack true ommatidia in the developmental and functional sense because the optically measured rhabdom density is up to four times higher than facet density in the frontal acute zone [34]. The close match in identified facet and rhabdom numbers in our study suggests that at least developmentally, the optical and receptive elements formed a single unit in the eye of hummingbird hawkmoths. Yet, functionally, they might not. The numerical match between rhabdom and facet numbers includes the facets at the rim of the compound eye, which might have a structural rather than functional role, as they were distinctly smaller than the main facets (figure 2*c*; electronic supplementary material, figure S4A), and covered by scales in the intact hawkmoths. Since these small facets comprised about 25% of the total facets (electronic supplementary material, figure S4A), if they are not optically active, there would be more rhabdoms than facets focusing light on them in the eye.

While our results thus support a general over-representation of rhabdoms to facets in the hummingbird hawkmoth eye, they do not represent the previously described increase in rhabdom density in the frontal eye (figure 3). On the contrary, rhabdoms were spaced more widely in the fronto-ventral part of the eye than the dorsal hemisphere (figure 3*a*). The denser rhabdom packing in the frontal eye observed previously using opthalmoscopic measurements might thus have been an optical effect. The rounded frontal cornea focusing light onto a very flat frontal retina could potentially produce a magnification of the focused image, leading to increased spatial resolution without a denser rhabdom packing. Future optical modelling will have to reveal whether this hypothesis holds, while developmental investigations might unravel how the highly inhomogeneous distribution of facet and rhabdom mosaics emerges.

### (c) Scaling of eye size compared to other insects

The scaling of the superposition eyes of hummingbird hawkmoths followed the same general trend described for the apposition eyes of other insect groups: they scaled negatively allometric with body size (bees [14–17], ants [18,19], butterflies [20,21] and flies [22]). The scaling exponent we observed in hawkmoths (average: 0.55) was slightly larger than in bumblebees (0.45) [17], and fell well within the ranges described for ants [18] and fruit flies [22]. Interestingly, head size scaled isometrically in the hawkmoths, thus resulting in proportionally smaller heads than eyes in smaller individuals. In line with this, overall brain size and optic lobe size also scales isometrically in this hawkmoth species [42], suggesting separate growth regulation for head and brain size on one hand, and eye size on the other hand.

The comparison of morphological structures related to visual sensitivity between hawkmoths and previously studied insects is of particular interest since the hawkmoths’ superposition compound eyes provide high visual sensitivity due to its specialized light-collecting optics [26,35]. We hypothesized that the trend to larger sensitivity in larger apposition compound eyes, as seen in bumblebees [15,17], would be less pronounced in the hummingbird hawkmoth, where sensitivity might be under less selection pressure because the superposition pupil increases light capture 200-fold [34,43]. Surprisingly, the opposite was the case: the allometric scaling exponent of the facet diameter with eye size was distinctly smaller than in bumblebees (0.71 [17]) and smaller than in fruit flies (0.57 [22]). The consequence of the relatively increased facet and rhabdom diameters, in combination with isometrically scaling focal and rhabdom lengths, was a distinctly increased ommatidial sensitivity in smaller eyes compared to isometric scaling (figure 4*a*). Thus, compared to insects with less light-sensitive apposition eyes [15,17,22], the highly sensitive superposition compound eyes of hawkmoths had the strongest optimization for single-ommatidium sensitivity.

It is important to note that our sensitivity estimation included an approximation of one important parameter of visual sensitivity: the rhabdom size. We estimated the scaling of rhabdom size by the scaling of the distance of neighbouring rhabdom centres in the retina, which represent an estimate of the size of neighbouring retinula units. This estimation has a justification in the particular morphology of the hawkmoth rhabdom, which spans the entire cross-section of each retinula unit [36], thus supporting the assumption that the rhabdom area directly scales with the size of the retinulae. Nevertheless, it is possible that the rhabdom morphology within the retinulae changes consistently in hawkmoth of different body size, thus affecting the scaling of visual sensitivity. To assess this quantitatively, the three-dimensional morphology of the rhabdoms of large and small hawkmoths would need to be reconstructed to investigate the allometric scaling of the precise rhabdom volume and correct the estimate of visual sensitivity accordingly.

### (d) Benefits of relatively increased facets and rhabdoms in superposition eyes

While the investment in high sensitivity in a diurnal species with highly sensitive eyes might be surprising, one needs to consider that increased facet and rhabdom diameters do not just support ommatidial sensitivity, but can also improve spatial acuity if the eye is diffraction limited [6–8]. The strongly negative allometric scaling of the facet diameter would reduce the size of single-facet based diffraction blur compared to isometric scaling (figure 4*c*). This scaling also results in relatively increased rhabdom diameters in small individuals, which further limits potential light-leakage effects into neighbouring ommatidia due to wave-guiding in the rhabdoms [13], because the rhabdom diameters remain several times larger than the wavelength of visible light (figure 3*c*). Light leakage is further prevented by the tracheal sheet around each photoreceptor unit [34].

While previous work suggests that the diffraction blur caused by a single facet in a compound eye linearly adds to the photoreceptor acceptance angle [10], and thus compromises spatial resolution, this assumption does not seem to hold for superposition compound eyes [44], nor indeed for apposition compound eyes [9,11]. In superposition compound eyes, the interaction of partially coherent light waves focused on a single rhabdom causes complex diffraction patterns that depend on the number of ommatidia in the superposition pupil [44]. This effect decreases the extent of the blur resulting from diffraction, and might thus release superposition compound eyes from the diffraction limitations on spatial acuity that are imposed by single facets. If this was indeed the case for hummingbird hawkmoth eyes, which future optical modelling studies need to confirm, the relatively enlarged facets in smaller hawkmoths might not contribute to improved spatial acuity by decreasing the half-width of the diffraction blur compared to isometric scaling (figure 4*c*).

It is furthermore important to consider that visual sensitivity does not just set the absolute detection limits of the eye but also determines how fine contrasts a visual system can resolve [6,7]. Thus, while sensitivity is high due to the eye design in hawkmoths, and these diurnal insects can still see [43] and perform visual behaviours even at moonlight intensities [45], the observed scaling might serve to maximize sensitivity for the purpose of retaining high contrast resolution in small hawkmoths. One benefit of high contrast sensitivity even for diurnal insects is the detection of small objects, which is ultimately restricted by the sensitivity of individual photoreceptive units [46]. Furthermore, high contrast sensitivity paired with high spatial resolution might be particularly adaptive for hovering insects, as it allows them to resolve motion cues both at slow hovering and fast forward flight speeds [47]. Thus, allometric scaling of facets and rhabdoms to retain high contrast sensitivity in small hawkmoths might provide benefits for spatial and motion tasks, on top of the high absolute sensitivity that their superposition compound eyes provide.

### (e) Optimizing eye performance across scales

One striking hypothesis for the observed scaling of the different optical structures emerged when we assessed its effect on the performance of the eye, compared to other possible scaling coefficients. The measured scaling exponents reduced the variation in sensitivity and spatial resolution across eye sizes, compared to isometric scaling. Indeed, they generated close to the minimum possible variation in eye parameter of all scaling factors, meaning that the eyes of larger and smaller hawkmoths varied the least possible in their spatial acuity and sensitivity (figure 4). This likely benefits the subsequent processing of visual information, because processing strategies can be largely retained across size ranges—particularly with respect to the processing that affects spatial resolution and visual sensitivity [36,48,49]. As discussed above, scaling that changes the contrast and spatial properties of the visual system might alter the perceptual thresholds for object or motion detection, for example, and thus require subsequent adjustments in the visual circuits to enable individuals of different sizes to successfully perform visual behaviours. Motion vision provides an interesting case because the spatial and temporal properties of the visual input are tightly entwined in the motion percept [50]. Consider, for example, two hummingbird hawkmoths with different body sizes, and thus with different spatial acuity due to allometric scaling, flying at the same speed in the same environment. Their neuronal responses to motion would be different, because motion-sensitive neurons are temporal frequency tuned, and the temporal frequencies they observed would differ depending on the spatial sampling base of their eyes [50]. How then would the motion vision system be adjusted to optimally code motion in the velocity range these insects experience—or is the adjustment performed behaviourally, so that moths with higher spatial acuity fly at lower speeds? Scaling the eye so that changes in spatial acuity and contrast sensitivity are minimized between large and small individuals, as observed in the hummingbird hawkmoth, will minimize the need for such behavioural or physiological adjustments, and thus markedly simplify the subsequent visual processing across body size ranges.

### (f) Adaptive consequences of eye scaling in solitary and social insects

The reduction of variation in sensitivity and acuity across hawkmoth sizes also suggests that larger and smaller hawkmoths would have similar visually driven behavioural abilities. In terms of spatial acuity, this is supported by recent findings, which show no difference in spatial resolution between large and small hawkmoths in an optic flow-based flight task [51]. Given that the estimated decrease in the photoreceptor acceptance angle in the smallest tested hawkmoths was only 15% lower than that in an 80% larger moth (figure 4*b*), and considering the range of spatial frequencies the hawkmoths responded to behaviourally [51], the lack of a behavioural phenotype might not be surprising. This is in stark contrast to bumblebees, where the spatial resolution improved by 30–50% (measured as the inter-ommatidial angle) in 50% larger bumblebees. Together with a distinct scaling of visual sensitivity with body size, these effects manifest in behaviour: larger bees forage at lower light intensities [24] and detect smaller point-targets than smaller ones [15,23]. In general, there might be a higher tolerance for variations in eye performance across scales in social insects, since the unit of selection is the colony [52], not the individual. In bumblebees, the workers that leave the nest to forage are typically larger individuals [53], so that a scaling of sensitivity benefits the colony in foragers with a higher sensitivity, while the smaller individuals can take up other tasks in the colony. In hawkmoths, where the unit of selection is the individual, a strong scaling of visual sensitivity with eye size would be maladaptive to a distinct proportion of the population, which might therefore have a lower tolerance for performance scaling with eye size. Future comparative work is required to resolve which role solitary lifestyle, phylogenetic heritage, and eye design play in the allometric scaling we found in hummingbird hawkmoths.

## Data Availability

All data analysed in this study are included in the manuscript and supporting data table file, and at https://doi.org/10.5061/dryad.44j0zpch4 [54]. All software packages used for analysis (in particular statistical analysis) are cited in the methods. The data are provided in electronic supplementary material [55].
